# Engaging Teenage Grassroots Soccer Players in Sports‐Related Concussion Education

**DOI:** 10.1111/hex.70813

**Published:** 2026-08-09

**Authors:** Abid Hussain, Dominic Malcolm

**Affiliations:** ^1^ Sir John Beckwith Centre for Sport, JB.2.07d, School of Sport, Exercise and Health Sciences Loughborough University Loughborough UK

**Keywords:** brain, concussion, education, head injuries, soccer injuries, sports injuries

## Abstract

**Introduction:**

Sports‐Related Concussion (SRC) presents potentially significant risks, particularly for teenage grassroots soccer players. Education is frequently proposed as a solution to address these risks; however, the effectiveness of educational interventions remains unproven, particularly for this demographic. This study aimed to explore how grassroots players' learning preferences can inform the design of educational interventions to mitigate SRC risks.

**Methods:**

An applied qualitative research design using Framework Analysis was employed to explore the lived experiences of 20 participants (15 males, 5 females) aged 14–18 years from a grassroots soccer club in an inner‐city UK setting. Focus groups were conducted to investigate learning preferences, delivery modes and engagement with SRC education. Analysis was informed by phenomenological principles to attend to participants' lived learning experiences.

**Results:**

Two overarching themes were identified from the analysis: sources of information and learning approaches. Participants predominantly accessed SRC information through short‐form video platforms, particularly TikTok and Instagram, highlighting the importance of familiarity and digital engagement. They expressed a preference for short, structured lessons, which may align with principles of cognitive load management and spaced learning. Hands‐on, visually supported instruction was favoured over didactic formats, aligning with experiential and multimedia learning perspectives. Message credibility was central; participants indicated that information was more likely to be accepted when delivered by trusted figures such as elite athletes or peers with lived experience of SRC, underscoring the social dimensions of learning.

**Conclusion:**

The findings suggest that SRC education for teenage grassroots soccer players could incorporate short, structured sessions; interactive and practical approaches; online quizzes, and the use of social media platforms and relatable information sources. The study points toward the value of co‐design with young athletes when developing contextually relevant educational approaches. However, as the research was conducted within a single grassroots soccer club, these design considerations should be interpreted as provisional and may not transfer directly across broader grassroots sporting environments. Further research is required to evaluate these approaches in practice.

**Patient or Public Contribution:**

Teenage grassroots soccer players were actively involved throughout the study. Participants contributed to intervention development by sharing learning preferences and experiences that informed structure, content and delivery methods. This participatory focus group approach helped to ensure that potential interventions reflected the target group's learning preferences. Public input was instrumental in shaping practical recommendations for SRC interventions and addressing historical limitations of SRC awareness programmes.

## 1 Introduction

Sports‐Related Concussion (SRC) is a form of traumatic brain injury caused by impulsive forces transmitted to the brain during sport or exercise [1]. Increasing worldwide media coverage and predominantly European research on SRC in professional soccer have raised public awareness of its potential long‐term effects [2, 3, 4]. Studies suggest that cumulative exposure to concussive and non‐concussive impacts, such as repeated heading, may increase the risk of neurodegenerative diseases [5, 6, 7], including Alzheimer's disease and chronic traumatic encephalopathy [8, 9].

In response to growing concerns regarding SRC, national governing bodies have introduced guidelines to prevent and reduce its incidence. In the United Kingdom, national concussion guidance for grassroots sport, including that adopted by the English Football Association (FA), emphasises the principle of ‘if in doubt, sit them out’ and places responsibility for recognising and managing SRC symptoms on players, coaches, volunteers and parents, while supporting a safe, self‐managed return to activity [10, 11]. The broader scope of participation reveals a vast grassroots landscape, where around 18,000 UK grassroots soccer clubs host regular play for thousands of teenagers [12]. Although the FA has implemented protocols to limit heading during training for children, there are no mandatory SRC education programmes for players at the grassroots level to mitigate SRC risks [11, 13].

Despite increased public awareness of SRC risks in youth sports, educational interventions have largely reflected a top‐down dissemination of medical knowledge [14]. These approaches often prioritise expert‐driven content over engagement with the specific concerns, lived experiences and needs of youth athletes and their communities [15]. Consequently, despite the proliferation of such initiatives across various sports, SRC remains a significant issue, and underreporting continues to be widespread [16].

Education remains a central component of national‐level concussion policies [10, 17]. However, reviews of SRC education programmes suggest that interventions typically improve factual knowledge related to injury prevention but have limited impact on long‐term behavioural change. Younger athletes also tend to perform worse than older athletes on post‐education assessments [18, 19, 20]. Moreover, the target population is rarely involved in intervention design, and when learning theory is used, it is typically selected by researchers rather than informed by engagement with the target community [15].

While education commonly increases knowledge of SRC signs and symptoms, these gains do not consistently translate into altered risk perception, reporting intentions or self‐removal from play [17]. This distinction is particularly salient within grassroots youth soccer, where limited medical supervision, restricted substitutions, volunteer coaching structures and strong peer dynamics may heighten pressures to continue playing despite injury [10]. Understanding how young athletes prefer to engage with and interpret educational content may therefore inform the development of SRC education that aims to improve knowledge, influence attitudes and support safer responses to SRC [20, 21].

Learning theories provide useful perspectives for understanding how teenagers engage with, process and apply educational information. Within grassroots SRC education, approaches that are developmentally appropriate, visually engaging, socially relevant and practically applied may better align with the learning experiences and preferences of teenage athletes. Four theoretical perspectives were considered relevant to this study. Cognitive Load Theory highlights the importance of presenting information in manageable ways to reduce overload and support attention [22]. Multimedia Learning and Dual Coding principles emphasise the value of combining verbal and visual information to support comprehension and recall [23, 24, 25]. Experiential Learning Theory highlights the role of practical and applied learning experiences in supporting understanding and engagement [26, 27]. Social Cognitive perspectives also draw attention to the influence of modelling, peer interaction and perceived credibility on learning and behaviour [28]. Together, these perspectives informed the exploration of how teenage grassroots soccer players preferred SRC education to be delivered and experienced.

The aim of this study was to explore how the learning experiences and preferences of teenage grassroots soccer players in the United Kingdom may inform the design and structure of SRC education. In doing so, the study addresses the limited involvement of young athletes in SRC education design and identifies provisional, theory‐informed design considerations to support future intervention development within grassroots soccer contexts.

## 2 Methods

This study employed an applied qualitative design using Framework Analysis to explore patterns in teenage grassroots soccer players' learning experiences relevant to SRC education. The analysis was informed by phenomenological principles, attending to participants' lived learning experiences, while maintaining a pragmatic, design‐oriented focus appropriate for applied health research. This approach recognises the influence of cultural, social, environmental and experiential factors in shaping how participants interpret and make meaning of their experiences [29, 30]. A qualitative design was selected to explore the complexity of participants' experiences and perspectives in greater depth. The study is reported in accordance with the Consolidated Criteria for Reporting Qualitative Research (COREQ) [31].

### 2.1 Participants

Participants were recruited through convenience and snowball sampling at a grassroots soccer club. The inclusion criteria specified males and females aged 14–18 years who were actively involved in competitive grassroots soccer. A total of 15 male and 5 female participants met these criteria and provided consent to participate. Participants spent a substantial amount of time engaged in soccer‐related activities each week, including two 2.5‐hour weekday training sessions and Sunday match fixtures, while also balancing academic commitments. The club was located in a relatively deprived inner‐city area and served a population of players who typically had limited financial resources. At the time of data collection, the club did not provide a formal SRC education programme for players or require completion of online SRC education. Prior SRC experiences emerged organically during focus group discussions but were not systematically recorded as participant characteristics. Similarly, playing position and detailed training exposure were not formally recorded. As a result, the findings may reflect the specific social, cultural and organisational characteristics of this grassroots soccer environment.

### 2.2 Ethics and Procedures

Ethical approval was obtained from Loughborough University's Research Ethics Committee 2023‐13062‐13057. Recruitment materials were distributed through existing club communication channels rather than direct individual approaches by the lead researcher. Written parental/guardian consent and participant assent were obtained prior to participation. Completed consent and assent forms were returned to the club welfare officer before data collection, providing separation between consent procedures and the lead researcher's clinical and sporting role within the club. Preliminary discussions were conducted with the participants to ensure they understood the purpose of the study, their rights to confidentiality and their ability to withdraw at any time without repercussions. Participation was voluntary, and participants were informed that participation or non‐participation would have no influence on team selection, playing time, medical care or their relationship with the club. To encourage participation, incentives were offered, including a £20 voucher for each participant and entry into a prize draw for a computer game.

### 2.3 Focus Groups

The 15 male participants were divided into three cohorts, each participating in three focus group sessions (Groups M1, M2 and M3), resulting in a total of nine sessions conducted between July 2023 and January 2024. The five female participants formed a single group (Group F1) and participated in three focus group sessions held between January and April 2024. Focus groups were organised by sex to reflect the club's existing team structure and to support group familiarity, comfort and open discussion. These were scheduled after the male groups due to initial recruitment challenges. Despite extended outreach efforts and flexible scheduling options, female participation remained limited. This may indicate a reluctance to engage, although the underlying reasons were not explicitly explored within the study. The study therefore reflects the experiences of a specific grassroots soccer setting with uneven gender representation, rather than attempting to capture the full diversity of teenage grassroots sport experiences.

Focus group allocation was based on participant availability and scheduling considerations while maintaining manageable group sizes to support discussion. Participants remained within the same focus group cohort across all three sessions. Sessions lasted between 15 and 32 minutes and were held at the grassroots club meeting room with the researcher (Author 1) and the club welfare officer present to support safeguarding procedures. Focus groups were facilitated by the lead researcher, who had prior clinical and applied experience working with adolescent athletes. While no formal facilitator training programme was undertaken specifically for this study, the lead researcher engaged in extensive preparation relating to qualitative interviewing and focus group facilitation methods and received ongoing supervisory support from the second author, an experienced qualitative researcher. All focus groups were audio‐recorded and transcribed verbatim. Analysis was conducted across all 12 sessions collectively, while also attending to areas of convergence, nuance and divergence between groups during interpretation.

Focus groups were employed due to their effectiveness in gathering data from multiple participants while fostering open discussions on sensitive topics [32]. A structured topic guide was used to explore three key areas: knowledge and experiences of SRC; behaviours, attitudes and reporting practices, and social interactions related to SRC. Discussions also explored how participants preferred SRC educational information to be delivered and experienced.

While the study was exploratory, the topic guide was informed by sensitising concepts drawn from the four learning theories previously discussed: Cognitive Load Theory, Multimedia/Dual Coding principles, Experiential Learning and Social Cognitive Theory. This ensured that prompts addressed lesson structure and pacing, preferred modalities (visual/video), practical learning approaches and the perceived credibility of different messengers. Insights relating to educational delivery and messaging were evident throughout the dataset. These included discussions surrounding preferred delivery methods, the role of digital and social media platforms, and the influence of credibility and social relationships on SRC messaging. Participants were encouraged to draw upon personal experiences across educational, sporting and social contexts. Although this paper focuses on selected aspects of the data, it is informed by the wider study's broader engagement with the SRC experiences of this community.

To preserve anonymity, the name and location of the soccer club are withheld. The data in this study are reported using focus group identifiers rather than attributing responses to individual participants. This approach safeguards personal identities while acknowledging the sensitive nature of the topics discussed [33]. To maintain authenticity, the data are presented verbatim, reflecting the participants' ages and educational levels [34].

### 2.4 Researcher Description

The lead researcher is a PhD candidate with 30 years of experience as a specialist sports podiatrist and 7 years of experience working with recreational soccer teams. In addition, the lead researcher is the head of sports medicine at the club at which the focus groups took place. The second author, who serves as the lead PhD supervisor, is an experienced qualitative researcher with an extensive publication record in SRC research. Both researchers contributed to the study design, data collection and analysis to enhance methodological rigour and uphold the integrity and trustworthiness of the study [35].

Prior to data collection, the researcher had an established relationship with participants through his role within the club, which may have influenced recruitment, participation and responses during discussions. While this familiarity may have supported rapport and openness, it also created the potential for socially desirable responses when discussing SRC behaviours and attitudes in a club setting. To minimise these influences, efforts were made to foster a relaxed, non‐judgemental environment, emphasise the exploratory nature of discussions and reassure participants that there were no right or wrong answers and that responses would not influence their sporting involvement or medical support within the club [36].

The lead researcher's dual role as a clinician and head of sports medicine was also recognised as a potential influence on power dynamics, participant engagement and data interpretation. Reflexive consideration was therefore given to role relationships throughout the study, particularly given that participants were adolescents discussing health‐related behaviours within their sporting environment. To reduce potential authority effects, the researcher adopted a facilitative rather than instructional stance during focus groups, emphasised the voluntary nature of participation and framed sessions as discussion‐based rather than evaluative. Reflexive discussions during analysis considered how the researcher's prior assumptions regarding SRC awareness, reporting behaviours and educational preferences may have influenced interpretation of the data. For example, participants' emphasis on peers, athletes and relatable messengers challenged the researcher's initial assumption that expert medical sources would be prioritised. This informed more open follow‐up questioning about credibility in later focus groups and encouraged greater caution when interpreting social media preferences as evidence of educational value. Ongoing supervisory dialogue and maintenance of a reflexive diary were used to critically examine how the researcher's positionality shaped data collection, analysis and theme development.

### 2.5 Data Analysis and Methodological Integrity

Data were analysed using Framework Analysis following the stages outlined by Gale et al. [37]. Focus group recordings were transcribed verbatim and repeatedly reviewed during familiarisation with the dataset. Initial semantic coding was conducted manually by the lead researcher, focusing on participants' explicit accounts relating to SRC learning experiences, educational preferences, delivery methods and social influences.

Codes were iteratively refined into a working analytical framework and charted into a framework matrix organised by focus group/session and key analytic categories, allowing comparison across participants, groups and sessions. Lower order themes were developed through the process of charting and comparing coded data within the matrix, before being refined into broader overarching themes. Through this process, patterns, relationships, tensions and areas of convergence and divergence were identified, contributing to further thematic refinement. Interpretive synthesis involved iterative movement between participant excerpts, thematic patterns and selected learning theory perspectives to generate provisional SRC education design considerations.

The second author reviewed the developing thematic structure, interpretations and manuscript drafts throughout the analytic process. Alternative interpretations and differences in analysis were discussed until consensus was reached.

Analytic memos were maintained throughout data analysis to document insights, interpretations and potential connections to enhance transparency and rigour. Reflexive memoing was also used to document how the lead researcher's existing relationship with the club and participants may have shaped interpretations of participant responses and emerging themes throughout the analytic process.

Reflexive practice was supported through maintenance of a reflexive diary, which enabled ongoing reflection on interpretive decisions throughout data collection and analysis. Across the 12 focus group sessions, recurring patterns and areas of variation were identified, providing sufficient analytic depth to support the development of coherent themes relevant to the study aim. The study was not designed to undertake formal comparative analysis across sex, age, educational stage or concussion history, although points of convergence and divergence across participant responses were considered during interpretation.

## 3 Results

Two overarching themes emerged from the data: Sources of Information and Learning Approaches. While broad patterns were identified across groups, some tensions and minority perspectives emerged regarding teaching approaches, lesson structure and source credibility. Discussions highlighted the influence of digital media, peers and teachers, alongside preferences for visual, practical and structured learning.

### 3.1 Sources of Information

When asked how they accessed news, the predominant response was ‘social media’. All participants engaged with social media, with estimated usage ranging from 1 to as many as 14 hours a day. Although this latter estimate should be interpreted cautiously, it nevertheless reflects the centrality of social media within participants' everyday routines. TikTok, Snapchat and Instagram Reels were preferred, while no participants reported using Facebook. A typical exchange illustrates this distinction when the interviewer asked, ‘Nobody uses Facebook?’ to which a participant responded, ‘No, my parents do, that's an adult thing’ (M3).

These platforms were valued for their video‐based and fast‐paced formats. When asked, ‘What kind of technology or social media do you use and how does that help you to learn?’ many replied, ‘Through videos’. Participants commented, ‘Yeah, I learn from TikTok videos’ (M2) and ‘I use Instagram Reels quite a bit’ (M2). Examples of information learned through TikTok included soccer techniques, recipes ‘to make cookies and pancakes’ (F1) and health‐related topics, with one participant stating that he used the platform to ‘Learn with videos on drugs and stuff’ (M2).

When the topic shifted to SRC awareness, TikTok was again a frequently mentioned source. One participant shared, ‘Yeah, I found a story on TikTok where someone got paralysed off it’ (F1). Another participant, when asked, ‘How did you learn about concussion?’ replied, ‘TikTok’ (M1). These examples highlight how such platforms may deliver information in ways that resonate with teenage soccer players. However, participants did not necessarily describe social media as inherently trustworthy or educationally superior. Rather, platforms such as TikTok and Instagram appeared to be valued primarily because they were familiar, fast‐paced and embedded within participants' everyday digital routines.

A lack of engagement with traditional media was also evident. When asked about reading newspapers, one participant stated, ‘Never in my life’ (M1). When prompted, ‘So how do you get your news about anything that's happening in the world?’ another participant replied, ‘Friends and social media’ (M1). Television was predominantly used to watch sports rather than for news. When asked about popular news channels, a participant stated, ‘I won't put [them] on the TV’ (M3) and ‘I watch zero news on TV’ (M1). Conversely, another participant stated, ‘you know what, the news, sometimes it comes on like TikTok’ (M2) and ‘I get my news from social media’ (F1). These responses suggest limited engagement with conventional news media unless the content was perceived as sufficiently engaging, such as sport‐related content.

Informal peer interaction emerged as a mechanism through which participants learned about soccer skills and SRC. One participant mentioned that if they needed clarification on something, they would approach the team captain, stating, ‘The captain, it's personal, but in training, I would ask him’ (M2). When asked if anyone had learned about SRC from another player, one participant responded, ‘Yeah, (participant name). But she does get head injuries from football as well, by heading the ball. She's the only one who headers the ball’ (F1). These responses highlight the importance of peer knowledge and leadership within teams.

Experiences with teachers varied, with many participants expressing dissatisfaction with formal education approaches. One participant noted, ‘If they're just like talking at me rather than talking to me, I don't understand it, you know what I mean?’ (M2). Another added, ‘The teacher doesn't explain it properly, so I get lost. If they'll ask you questions throughout, then you're more engaged as well, so you can understand’ (M3).

The perceived credibility of information sources appeared important in shaping engagement with SRC messaging. Participants expressed a clear preference for information delivered by figures perceived as relatable or experienced, emphasising that famous soccer players such as Kylian Mbappé would have significant influence. One participant remarked, ‘He's a big influence right now… he's all over FIFA. All over football. He's the next big thing basically, and if he says something, people are more likely going to listen’ (M3). This demonstrates how the perceived relevance and authority of a figure can enhance the impact of a message. Similarly, participants valued testimonies from individuals who have direct experience with SRC, stating, ‘Probably a teammate that's had concussion will know more’ (M1) and suggesting a combination of expertise and relatability by adding, ‘Doctor and teammate that's had concussion’ (M1). These insights imply that SRC messaging may be more engaging when delivered by credible and relatable individuals. This approach aligns with strategies used in SRC interventions across other populations [14], but has rarely been explored with teenage soccer players.

### 3.2 Learning Approaches

Participants consistently valued a combination of visual and practical learning methods. While all expressed dissatisfaction with learning delivered solely through PowerPoint presentations, some adopted a more context‐dependent perspective, suggesting that these approaches could be effective when delivery style aligned with individual learning preferences:

I think in school, the way you learn, I think it depends on the style of the teacher, so if they're just reading stuff to you off the PowerPoint then it's not good, but if the way that they're presenting it to you and the way that they're teaching you, it suits your learning, then obviously you're going to remember it a bit more and you just kind of adapt to it easier.

(M1)

This indicates that participants were not uniformly opposed to teacher‐led instruction itself, but rather to passive or poorly delivered forms of communication that limited interaction and engagement.

Several participants preferred learning through trial and error; ‘Yeah, practice makes perfect, doesn't it?’ (M2). Another noted, ‘Like the best way to improve because then like practising’ (M1). When discussing learning in school, participants described the value of making mistakes and practising repeatedly until concepts were understood. This emphasis on repetition suggested that persistence and learning through mistakes supported understanding and mastery (M2):

Interviewer: How do you like to learn in school? Do you like to learn by getting something wrong a lot of times and then finally having that moment where you think, yeah, I understand it?

Participant: Yes

Participant: Just repeating the same thing

Participant: Practising over and over again, until I understand, it works

Another exchange revealed participants' positive attitudes toward learning through online quizzes. Interactive quizzes were described as enjoyable and engaging learning approaches. Enthusiasm for quizzes also extended to sport‐specific applications, with one participant noting, ‘That would be brilliant, yeah, a quiz to test our concussion knowledge during the season’ (M2), indicating the potential of such tools to support SRC education.

When asked about their preferred learning approach, the vast majority of participants expressed a strong preference for practical, hands‐on methods over theoretical instruction, such as board‐based teaching. Participants felt practical demonstrations improved understanding and memory retention. As one participant emphatically stated, ‘Practically. Practically. Practically… It's easier to understand’ (F1). Others added, ‘I'd rather him do a practical example and use props’ (M1) and ‘It makes it visual’ (M2).

This preference for hands‐on learning extended to the context of SRC education. When asked what type of practical demonstration would help them better understand SRC, one participant suggested, ‘Practical dummy of how a brain moves in a skull, like a jelly in a box’ (M3). Building on this perspective, another participant explained, ‘Yeah, like imagine when you header a ball, yeah, and like we don't see what our brain does’ (M3). These preferences were also reflected in the following exchange (F1):

Interviewer: When your coach teaches you something on the pitch, how do you find it best for him to teach you, for you to learn? Is it on a board, is it out on the pitch practically? How do you like to learn?

Participants (all): Practically.

Interviewer: Why?

Participant: Because it makes sense, it's easier to understand and remember what being taught when it's practical

Interviewer: What are the best kind of lessons you've ever had in school or college?

Participant: Sports and science.

Participant: Practical.

Interviewer: Give me an example?

Participant: Science practicals

Participant: We always look forward to the practicals.

Preferences regarding lesson duration and structure centred on shorter, more manageable SRC sessions. Participants valued shorter, more frequent sessions to avoid information overload and support retention (M1):

Interviewer: Anything else that you think that would be helpful in an intervention for concussion? Any ideas?

Participant: What, with the lesson?

Interviewer: Yeah, for the lesson.

Participant: It can't be too long

Interviewer: Can't be too long?

Participant: Just make it short

Interviewer: How short?

Participant: Probably about ten minutes, a quick ten‐minute lesson but more often, if that makes sense?

Interviewer: Ten‐minute lesson but often?

Participant: Yeah

Interviewer: Anybody else?

Another Participant: A bit longer than that I'd say.

Interviewer: How long would you say?

Another Participant: I'd say half an hour, I don't want all the information in one go if you know what I mean

Although shorter lessons were generally preferred, views differed regarding appropriate duration. Participants also described challenges associated with lengthy explanations and rushed teaching. One participant explained, ‘Your brain has to process it and the teachers just presenting something, they're basically rushing you. I want the information slowly’ (F1). Another highlighted the confusion caused by poor explanations: ‘If you don't understand what the teacher is saying, you're lost’ (M2). To address these challenges, participants emphasised incremental, ‘step‐by‐step’ approaches that avoided presenting ‘too much in one go’ (M2). Repetition and spaced delivery across ‘multiple sessions’ (M3) were also perceived to help information ‘stick’ (M3) more effectively than intensive single sessions. These insights highlight the potential value of manageable, structured and repeated learning within SRC education.

Interactive lesson structures and active participation were consistently viewed as important components of effective learning. When asked what makes a good lesson, participants repeatedly stated, ‘Interactive’, adding that it should be ‘fun’ and ‘engaging’. This contrasted sharply with their description of the worst lesson, where a participant recalled, ‘The guy just gave us [a] book to look at without even explaining it, I didn't learn jack [anything]’ (M2). Another stated that a teacher had tried to ‘explain it to me like I'm an adult but I'm not’ (M3), illustrating their dislike for age‐inappropriate communication and passive, unstructured teaching methods.

The importance of clear learning objectives was also emphasised, with participants agreeing that being told the focus of a lesson beforehand would enhance engagement. One participant explained, ‘Yeah, because it would give you direction’ (M3), suggesting that structured guidance may support attention and engagement.

## 4 Discussion

The findings are interpreted through four complementary learning theory perspectives: Cognitive Load Theory, Multimedia/Dual Coding principles, Experiential Learning and Social Cognitive Theory, which are used selectively to interpret key themes emerging from participants' accounts. It should be noted, however, that this study identifies reported learning preferences and design considerations; it does not demonstrate that these approaches improve SRC knowledge, reporting behaviour or safety outcomes.

Although broad patterns emerged across the dataset, some variation and nuance were evident. For example, while many participants expressed dissatisfaction with traditional classroom approaches, others differentiated between passive instruction and engaging teacher‐led delivery. Similarly, although social media platforms were widely preferred due to familiarity and accessibility, participants did not necessarily present these platforms as more trustworthy or educationally effective. These tensions highlight the importance of avoiding uniform assumptions regarding teenage learning preferences within grassroots sport contexts. Focus group responses offered valuable insights into how teenage grassroots soccer players engage with different information sources and educational approaches. Visual, practical and interactive formats were consistently preferred. These insights may inform SRC education within comparable grassroots soccer contexts by highlighting participant‐reported content preferences, delivery formats and engagement strategies.

The preference for interactive and practical learning reflected a desire for understanding through doing rather than passive information reception. The use of demonstrations and props may be interpreted through experiential learning perspectives, which emphasise applied and reflective engagement with learning tasks [26]. For example, using a ‘jelly brain in a box’ to demonstrate how the brain moves inside the skull during an SRC may help make abstract SRC concepts more concrete and accessible for teenage players.

Dislike of PowerPoint presentations appeared to reflect not a rejection of formal instruction, but a preference for visually rich, multimodal content that supports comprehension and engagement. Multimedia learning principles provide one explanation for these preferences and suggest strategies to address limitations of text‐heavy instructional formats. By incorporating vibrant colours, appealing fonts and integrated multimedia elements such as videos, interactive PowerPoint presentations may better align with teenage learners' reported preferences for visually engaging content. These preferences may also be interpreted through Dual Coding Theory [25], which proposes that combining verbal and visual information can support comprehension and recall [38].

Preferences for shorter, repeated SRC education sessions appeared linked to concerns about information overload and maintaining attention during learning. These views may align with educational perspectives that emphasise cognitive load management and the spaced reinforcement of information over time [39]. Incorporating perspectives of the target demographic through a co‐design process could help guide structuring of interventions into brief, repeated sessions intended to reduce cognitive overload while supporting reinforcement of key information. Lessons lasting between 10 and 30 minutes were preferred, with content delivered repeatedly across sessions throughout the soccer season. This could inform lesson planning by aligning educational delivery with participants' expectations and capacities and may be perceived as more manageable than longer, information‐heavy sessions [40].

Enthusiasm for quizzes and gamified learning suggests that active, feedback‐oriented formats may be perceived as more engaging than passive instruction. This may be interpreted through educational literature indicating that interactive and gamified approaches may support learner engagement and participation [41, 42, 43]. Online SRC quizzes delivered across the season could provide opportunities for repetition and reflection, although their influence on SRC‐related learning and behaviour remains uncertain.

Social media platforms were described as familiar, accessible and visually engaging environments through which participants commonly encountered information. These findings suggest opportunities for delivering SRC content through formats that align closely with participants' existing digital habits. These preferences may reflect the media habits, social dynamics and educational experiences of this specific participant group rather than teenage athletes more broadly. Participants favoured platforms such as TikTok and Instagram for their fast‐paced, visually engaging formats. These platforms may provide accessible channels for SRC education, for example through short myth‐busting videos addressing common misconceptions about SRC, peer‐led testimonials describing decisions to self‐remove from play or brief animated demonstrations illustrating how the brain moves within the skull during impact. Drawing on participants' insights, researchers could co‐create credible, culturally relevant SRC content tailored to teenage soccer players' preferred digital environments. Scenario‐based content or interactive quizzes delivered across the soccer season may reinforce key messages while aligning with teenagers' media habits [44]. However, participants' use of TikTok and Instagram likely reflects a preference for familiar and engaging formats rather than evidence that these platforms improve comprehension, trust or behaviour change. Although club‐ or organisation‐led social media approaches may offer greater oversight and credibility, careful governance of content and messaging would still be important to minimise the potential influence of misinformation or overly simplistic messaging.

The perceived credibility of messengers highlighted the social and relational dimensions of learning, with particular influence attributed to figures perceived as relatable or experienced, such as peers, team captains and athletes with lived SRC experience. These findings highlight the potential importance of involving stakeholders such as players and coaches in both the design and dissemination of SRC education. Social learning perspectives may help explain why participants perceived these individuals as more influential and relatable sources of information [45, 46]. Providing one‐to‐one teaching sessions for team captains of each age group before introducing the intervention to the full squad could further strengthen this approach, as captains can act as influential peer leaders within their teams. By empowering captains to introduce and advocate for SRC education, the intervention may gain a more relatable channel for information dissemination, although this would require evaluation [47]. This approach aligns with previous research showing that athletes with lived experience of SRC may enhance the relatability and perceived relevance of concussion messaging among their peers [14]. The insights of participants reflect the value of engaging the target audience in the co‐design process, potentially enhancing the relatability and relevance of educational content [48]. While education plays a critical role in improving awareness and engagement, it is unlikely to be sufficient on its own to change SRC reporting behaviours without alignment with broader social, cultural and organisational factors within grassroots sport [17].

### 4.1 Limitations

The findings should be interpreted in light of several limitations. The study was conducted within a single grassroots soccer club located in a relatively deprived inner‐city area of the United Kingdom, and the findings may therefore reflect the specific social, cultural and organisational characteristics of this environment. In addition, the sample was characterised by a gender imbalance, with the majority of participants identifying as male. The lead researcher's established role at the club may have influenced participant disclosure and interaction during focus groups despite efforts to minimise authority effects and encourage open discussion. Participants may have moderated responses relating to SRC behaviours, reporting practices or attitudes due to the researcher's clinical and organisational role within the club environment. Some educational design preferences appeared similar across male and female participants; however, the limited female representation means these observations should be interpreted cautiously. The framing and delivery of SRC messaging should therefore remain sensitive to the social norms and team cultures within which education is delivered. The single‐site design also limits the transferability of findings to other grassroots sporting contexts, including rural clubs, higher resourced environments, alternative sporting cultures or settings with different coaching structures and educational experiences.

Consistent with qualitative research principles, the aim of this study was not statistical generalisation but analytic transferability [35]. The contextual detail provided enables readers to assess the transferability of the findings to comparable teenage grassroots team sport settings operating within similar organisational, social and cultural contexts in the United Kingdom.

Importantly, this study explored stated learning preferences rather than observed educational effectiveness. Preferences for short sessions, practical demonstrations, quizzes or social media delivery should not be interpreted as evidence that these approaches improve SRC knowledge, reporting behaviour or safety outcomes. Future intervention studies are needed to test whether SRC education designed around these participant‐informed approaches produces measurable changes in understanding, attitudes or behaviour. Similarly, interest in social media‐based delivery may reflect familiarity and accessibility rather than educational effectiveness, trustworthiness or comprehension.

Although some nuanced and minority perspectives were identified, the sample size and qualitative design did not permit robust subgroup comparisons across variables such as sex, age, educational context or prior SRC experience. Future research should examine whether similar learning preferences emerge across different grassroots clubs, sporting contexts, geographical regions and demographic groups.

## 5 Conclusion

To our knowledge, this study is among the first to explore grassroots teenage soccer players' learning preferences in relation to SRC education. By embedding player‐derived insights within established learning theories, this study offers provisional design considerations for context‐specific SRC education in community sport. These considerations may inform the future development and evaluation of SRC educational interventions within comparable grassroots teenage soccer contexts.

- Short and Structured Lessons: Brief, repeated sessions may help align SRC education with participants' preferences for manageable and less cognitively demanding learning formats.
- Interactive and Practical Learning: Practical demonstrations and props may help make SRC concepts more concrete and accessible for teenage players.
- Incorporating Multimedia: Videos and quizzes may support engagement, repetition and reflection, although their impact on SRC knowledge retention and behavioural outcomes requires further evaluation.
- Leveraging Social Media: Platforms such as TikTok and Instagram may provide familiar and accessible routes for delivering SRC content, provided issues of credibility and misinformation are carefully considered.

- Credible and Relatable Sources: Athletes, peers and trusted figures with lived SRC experience may enhance the perceived relevance and acceptability of educational messaging, although their influence on reporting behaviour warrants further study.

To inform the development of these strategies, it is important to acknowledge that children and teenagers learn in varied and complex ways. Designing SRC programmes with this diversity in mind, rather than focusing exclusively on the transmission of expert‐defined content, could enhance developmental appropriateness, engagement and overall impact. By grounding educational interventions in established theories of learning, programme design may more closely reflect the ways in which young people acquire, process and retain knowledge.

These recommendations should be understood as hypothesis‐generating and intended to inform future co‐produced intervention development and evaluation rather than as prescriptive or definitive solutions. Drawing on participants' insights, the study highlights the potential importance of engagement, accessibility, credibility and cultural relevance in SRC education for teenage grassroots soccer players. This approach supports context‐sensitive intervention design while allowing educational strategies to remain responsive to both learning theory perspectives and the lived experiences of the target population.

Although previous SRC education initiatives have shown inconsistent behavioural impact, education remains important in grassroots settings where medical support and regulatory enforcement may be limited. Grounding SRC education in learning theory and involving teenage athletes in content development may enhance relevance, engagement and perceived acceptability. Rather than demonstrating intervention effectiveness, this study offers theory‐informed and player‐informed design considerations that can be evaluated and refined through future SRC education research across diverse grassroots sporting environments.

## Author Contributions

**Abid Hussain:** conceptualization, investigation, methodology, validation, writing – review and editing, writing – original draft, visualization, formal analysis. **Dominic Malcolm:** conceptualization, writing – review and editing, supervision, validation, formal analysis.

## Funding

The authors have nothing to report.

## Conflicts of Interest

The authors declare no conflicts of interest.

## Data Availability

The data that support the findings of this study are available from the corresponding author upon reasonable request.
